# Exploring the Complex Connection Between Diabetes and Cardiovascular Disease: Analyzing Approaches to Mitigate Cardiovascular Risk in Patients With Diabetes

**DOI:** 10.7759/cureus.43882

**Published:** 2023-08-21

**Authors:** FNU Jyotsna, Areeba Ahmed, Kamal Kumar, Paramjeet Kaur, Mitul Hareshkumar Chaudhary, Sagar Kumar, Ejaz Khan, Bushra Khanam, Syeda Urooba Shah, Giustino Varrassi, Mahima Khatri, Satesh Kumar, Kishan Ashokbhai Kakadiya

**Affiliations:** 1 Medicine, Dr. B. R. Ambedkar Medical College & Hospital, Mohali, IND; 2 Medicine, Fatima Jinnah Medical University, Lahore, PAK; 3 Medicine, Chandka Medical College, Larkana, PAK; 4 Internal Medicine, Guru Gobind Singh Medical College, Faridkot, IND; 5 Medicine, AMA School of Medicine, Makati, PHL; 6 Dermatology, All India Institute of Medical Sciences, New Delhi, New Delhi, IND; 7 Internal Medicine, National Tuberculosis Control Center, Kathmandu, NPL; 8 Medicine, Jinnah Sindh Medical University, Karachi, PAK; 9 Pain Medicine, Paolo Procacci Foundation, Rome, ITA; 10 Medicine and Surgery, Dow University of Health Sciences, Karachi, PAK; 11 Medicine and Surgery, Shaheed Mohtarma Benazir Bhutto Medical College, Karachi, PAK; 12 Medicine, Government Medical College, Surat, Surat, IND

**Keywords:** treatment options, preventive measures, insulin resistance, cardiovascular risk, cardiovascular disease (cvd), diabetes

## Abstract

Cardiovascular disease (CVD) is the primary cause of morbidity and mortality in individuals diagnosed with diabetes mellitus. This narrative review offers a comprehensive examination of the complex correlation between diabetes and cardiovascular complications. The objective of this review is to analyze the most recent evidence on preventive measures and treatment options for mitigating cardiovascular risk in patients with diabetes, by synthesizing existing literature. Insulin resistance plays a crucial role in connecting diabetes and CVD, leading to the development of dyslipidemia and atherogenesis. As a result, the risk of cardiovascular events in individuals with diabetes is significantly elevated. Moreover, the presence of hyperglycemia-induced oxidative stress and inflammation serves to intensify endothelial dysfunction and vascular damage, thereby exacerbating the risk of cardiovascular complications. The interaction between diabetes and CVD frequently speeds up the development of atherosclerotic plaque, making the plaque more prone to rupture. This can lead to severe cardiovascular events such as myocardial infarction and stroke. It is crucial to comprehend the intricate relationship between diabetes and CVD in order to formulate effective strategies aimed at enhancing patient outcomes and mitigating the burden associated with these interconnected chronic conditions. Healthcare practitioners can enhance the quality of life and reduce mortality rates associated with CVD in diabetic patients by thoroughly examining evidence-based preventive measures and treatment options. This approach allows them to make informed decisions when managing cardiovascular risk. In summary, this narrative review provides a valuable resource for healthcare professionals and researchers, presenting a comprehensive analysis of the complex relationship between diabetes and CVD. By providing a comprehensive analysis of the latest evidence and elucidating the underlying mechanisms, this review seeks to establish a foundation for the development of innovative strategies in diabetes management. These strategies have the potential to significantly improve cardiovascular outcomes and enhance overall patient care.

## Introduction and background

Diabetes mellitus is a long-term metabolic problem that causes high blood glucose levels because the body cannot make or use insulin properly. It has become a widespread epidemic that affects millions of people all over the world. According to the International Diabetes Federation (IDF), about 463 million people aged 20-79 had diabetes in 2019, and this number is expected to rise to 700 million by 2045 [1]. Diabetes does not just cause high blood glucose levels; it also causes a number of long-term problems that have a big effect on the health and well-being of people with it. One of the most worrying things about diabetes is that it can lead to cardiovascular disease (CVD). Cardiovascular illnesses, such as coronary artery disease, heart failure, and stroke, are major causes of death and disability in people with diabetes. Research has shown over and over again that people with diabetes are two to four times more likely to get CVD than people without diabetes [2]. This higher risk is caused by a number of things, such as insulin resistance, dyslipidemia, high blood pressure, and systemic inflammation, which all help atherosclerosis, the underlying cause of most cardiovascular events, grow and get worse. Diabetes also makes endothelial dysfunction, oxidative stress, and abnormal platelet function worse, which makes the chance of heart problems even higher [3]. Also, having both diabetes and CVD often makes things worse, making it more likely that the same thing will happen again and making healthcare costs go up [4]. Because of this, the relationship between diabetes and CVD has important clinical effects, and it is important to find effective ways to manage cardiovascular risk in diabetic patients. Because diabetes is a big problem and has a big effect on cardiovascular health, it is important to look at all the data about how to manage cardiovascular risk in diabetic patients. Over the years, different ways of treating this vulnerable group have been looked into to improve outcomes and lower the chance of CVD.

Glycemic control, which aims to keep blood glucose levels within a goal range, is a key part of taking care of diabetes. It has been shown that intensive control of blood sugar lowers the chance of microvascular problems like nephropathy and retinopathy [5]. But the evidence about its effect on cardiovascular outcomes has been mixed. Some studies have shown a small decrease in cardiovascular events, while others have raised worries about possible harm from hypoglycemia and weight gain caused by some diabetes drugs [6]. In the last few years, there has been a shift in how diabetic patients with cardiovascular risk are treated, with more attention being paid to multifactorial intervention methods. These methods are called the "ABCs" of diabetes care because they try to treat not only hyperglycemia but also high blood pressure, high cholesterol, and being overweight or obese. Several important clinical studies have shown that aggressive management of risk factors can lower the number of cardiovascular events in people with diabetes. For instance, the Empagliflozin Cardiovascular Outcome Event Trial in Type 2 Diabetes Mellitus Patients (EMPA-REG OUTCOME) and the Canagliflozin Cardiovascular Assessment Study (CANVAS) have shown that sodium-glucose cotransporter-2 (SGLT-2) inhibitors, a class of antidiabetic agents, not only improve glycemic control but also confer cardiovascular benefits, including a reduction in the risk of heart failure and cardiovascular death [7-9]. Trials looking at the effects of glucagon-like peptide-1 receptor agonists (GLP-1 RAs), such as the Liraglutide Effect and Action in Diabetes: Evaluation of Cardiovascular Outcome Results (LEADER) and the Trial to Evaluate Cardiovascular and Other Long-term Outcomes with Semaglutide in Subjects with Type 2 Diabetes (SUSTAIN-6), have shown that cardiovascular risk is lower in diabetic patients [10]. Based on these new results, it is clear that diabetic patients need a full narrative review of how to deal with cardiovascular risk. This review aims to give healthcare professionals a full understanding of the latest evidence-based strategies to improve cardiovascular outcomes in diabetics. It does this by analyzing the available literature and incorporating the results of relevant clinical trials.

## Review

Methodology

The study "Exploring the Complex Connection Between Diabetes and Cardiovascular Disease: Analyzing Approaches to Mitigate Cardiovascular Risk in Patients With Diabetes" utilized a rigorous and thorough methodology to examine the intricate correlation between diabetes and CVD. The study specifically concentrated on identifying strategies to decrease cardiovascular risk in individuals with diabetes. To commence the literature search, a meticulously curated assortment of esteemed academic databases and search engines was employed. Thorough investigations were conducted on reputable sources, including PubMed, MEDLINE, Embase, and Google Scholar. These databases were selected based on their comprehensive coverage of medical and scientific literature pertaining to the subject matter, thereby facilitating a thorough investigation of the research topic.

The search strategy utilized a combination of carefully selected keywords and controlled vocabulary terms (MeSH terms) relevant to diabetes, CVD, cardiovascular risk reduction, and interventions aimed at individuals with diabetes. The utilization of Boolean operators such as AND and OR has improved the accuracy of the search outcomes, facilitating the identification of studies that directly pertain to the research objectives. After conducting the initial search, a methodical screening process was implemented. The obtained articles underwent a thorough screening process consisting of two steps. The titles and abstracts were initially examined to assess their alignment with the research focus and inclusion criteria. A comprehensive review was conducted of the chosen articles' complete texts to evaluate their pertinence, caliber, and impact on the research inquiry.

A comprehensive analysis was conducted on 30 articles as part of this systematic process. For detailed information regarding the number of reports reviewed, search methodology, databases utilized, search terms employed, and the stepwise procedure of article selection, please refer to the methodology section of the research paper. This section presents a comprehensive and transparent overview of the study's methodology, highlighting the measures taken to ensure the validity and comprehensiveness of the findings. The study's methodology demonstrated a systematic and thorough approach to investigating the complex relationship between diabetes and CVD. Additionally, it examined strategies aimed at reducing cardiovascular risk in patients with diabetes. The systematic methodology employed in this study and the comprehensive review of 30 pertinent articles enhanced the depth and credibility of the analysis pertaining to this intricate correlation.

Mechanisms linking diabetes and CVD

Insulin Resistance and Atherogenic Dyslipidemia

Insulin resistance is a prominent characteristic of type 2 diabetes mellitus and a substantial determinant of CVD risk. Insulin resistance is characterized by a reduced responsiveness of specific tissues, including muscle, liver, and adipose tissue, to the physiological effects of insulin. This results in impaired glucose uptake and metabolism. Insulin resistance is known to play a role in the disruption of lipid metabolism and the emergence of atherogenic dyslipidemia. This condition is characterized by elevated triglyceride (TG) levels, decreased high-density lipoprotein cholesterol (HDL-C) levels, and an increase in small, dense low-density lipoprotein cholesterol (LDL-C) particles. It is essential to have a comprehensive understanding of the complex connection between insulin resistance and atherogenic dyslipidemia in order to effectively manage cardiovascular risk in patients diagnosed with diabetes. Insulin plays a crucial role in lipid metabolism through the regulation of key enzymes and transporters involved in lipid synthesis, storage, and clearance. In tissues that are sensitive to insulin, such as muscle and adipose tissue, the hormone insulin facilitates the uptake of glucose and hinders the breakdown of lipids, resulting in a decrease in the release of free fatty acids (FFAs) into the bloodstream. As a result, insulin plays a crucial role in regulating and reducing the levels of circulating FFAs. This is of significant importance as elevated FFAs have been linked to insulin resistance and disruptions in glucose homeostasis [11]. In contrast, insulin promotes lipogenesis in the liver by facilitating the conversion of FFAs and glucose into TGs. The TG-rich very-low-density lipoproteins (VLDLs) are subsequently released into the bloodstream, thereby contributing to hypertriglyceridemia, which is a defining characteristic of atherogenic dyslipidemia [11,12]. In cases of insulin resistance, the liver persists in generating an excessive amount of VLDL particles, which worsens the condition of elevated levels of triglycerides in the blood and the buildup of small, dense LDL-C particles.

The presence of atherogenic dyslipidemia in individuals with insulin resistance: Atherogenic dyslipidemia is characterized by increased levels of plasma TGs, decreased levels of HDL-C, and a shift toward a predominance of small, dense LDL-C particles. The lipid profile presented in this case demonstrates a notable elevation in risk for developing atherosclerosis and experiencing cardiovascular events, such as myocardial infarction and stroke [13]. Hypertriglyceridemia is a recognized characteristic of insulin resistance and plays a pivotal role in the progression of atherogenic dyslipidemia. In conditions of insulin resistance, adipose tissue experiences reduced efficacy in storing surplus FFAs, resulting in an elevated release of FFAs into the bloodstream. The heightened accessibility of FFAs within the liver facilitates the synthesis and release of VLDLs, resulting in elevated levels of TGs in the bloodstream [12,14]. In addition, it should be noted that insulin resistance has an impact on the metabolism of high-density lipoprotein (HDL) particles. It is well established that insulin has the ability to enhance the synthesis of apolipoprotein A-I, a key structural protein found in HDL particles. However, in states of insulin resistance, this physiological process becomes impaired, resulting in decreased levels of HDL-C [11]. HDL particles are essential components in the process of reverse cholesterol transport. This process involves the removal of excess cholesterol from peripheral tissues and its transportation to the liver for excretion. Decreased levels of HDL-C compromise the integrity of this protective mechanism, thereby contributing to the advancement of atherosclerosis. In addition, insulin resistance has the effect of modifying the composition of low-density lipoprotein (LDL) particles, resulting in the creation of small, dense LDL-C particles that possess a higher degree of atherogenicity compared to larger, buoyant LDL particles. Insulin is responsible for regulating the activity of enzymes involved in the metabolism of LDL particles, including cholesterol ester transfer protein (CETP) and hepatic lipase. These enzymes are involved in the conversion of LDL particles into smaller, denser forms [15]. The presence of small, dense LDL-C particles in the bloodstream leads to an extended duration within the arterial wall and heightened vulnerability to oxidation. This, in turn, contributes to the progression of atherosclerotic plaques.

Clinical implications and interventions: The correlation between insulin resistance and atherogenic dyslipidemia underscores the significance of implementing comprehensive management strategies in individuals with diabetes to effectively reduce cardiovascular risk. Therapeutic interventions focused on enhancing insulin sensitivity and lipid metabolism can yield substantial advantages in mitigating cardiovascular events. In the management of insulin resistance and dyslipidemia, it is crucial to incorporate lifestyle modifications such as engaging in regular physical activity and making dietary changes. Research studies have demonstrated that weight loss and physical exercise can effectively enhance insulin sensitivity and improve lipid profiles. These improvements are achieved by reducing plasma TG levels and increasing high-density lipoprotein cholesterol (HDL-C) levels [12,13]. Pharmacological interventions are essential in the management of atherogenic dyslipidemia in patients with insulin resistance. Statins, a pharmacological class of lipid-lowering agents, are widely regarded as the fundamental component in the management of dyslipidemia. Extensive research has consistently demonstrated their ability to effectively decrease levels of LDL-C and substantially mitigate the risk of cardiovascular events. In addition, recent advancements in lipid-lowering medications, such as PCSK9 inhibitors, have shown significant reductions in LDL-C levels and cardiovascular advantages, especially in patients with ongoing hypercholesterolemia despite treatment with statins [14]. In recent years, there has been an increased recognition of the significance of antidiabetic medications that exhibit favorable effects on lipid metabolism in the management of cardiovascular risk. For example, it has been demonstrated that GLP-1 receptor agonists and SGLT-2 inhibitors, in addition to their ability to lower glucose levels, have the potential to enhance atherogenic dyslipidemia and decrease cardiovascular events in individuals diagnosed with type 2 diabetes [9,10]. These agents have demonstrated promising effects in reducing plasma TG levels, increasing HDL-C levels, and facilitating the formation of larger, less atherogenic LDL particles.

The association between insulin resistance and atherogenic dyslipidemia is closely intertwined and plays a significant role in the heightened cardiovascular risk observed in individuals with diabetes. It is imperative to comprehend the complex interplay among insulin resistance, lipid metabolism, and dyslipidemia in order to devise precise therapeutic approaches that can effectively mitigate cardiovascular risk. Implementing comprehensive management strategies that effectively target both glucose and lipid abnormalities has the potential to significantly decrease the occurrence of cardiovascular events. This, in turn, can greatly enhance the long-term outcomes and overall quality of life for individuals living with diabetes.

Hyperglycemia and Oxidative Stress

Hyperglycemia, which is characterized by elevated levels of blood glucose, is a defining characteristic of diabetes mellitus. It is linked to various complications such as CVD, neuropathy, nephropathy, and retinopathy. Oxidative stress is a significant mechanism that underlies the pathophysiology of complications associated with hyperglycemia. Oxidative stress is characterized by a disruption in the equilibrium between the generation of reactive oxygen species (ROS) and the body's capacity to counteract them with antioxidant defense mechanisms, leading to detrimental effects on cellular integrity. The complex relationship between elevated blood sugar levels (hyperglycemia) and the harmful effects of oxidative stress has been thoroughly investigated and holds great significance in clinical practice.

The relationship between ROS production and mitochondrial dysfunction: Hyperglycemia induces the upregulation of ROS generation through multiple mechanisms, such as the mitochondrial electron transport chain, the formation of advanced glycation end products (AGEs), and the activation of NADPH oxidase. Mitochondrial dysfunction, which frequently occurs as a result of prolonged hyperglycemia, additionally contributes to the generation of ROS, thereby establishing a detrimental cycle of oxidative stress [15].

Inflammation and oxidative stress: Elevated blood glucose levels stimulate inflammatory pathways, resulting in heightened synthesis of pro-inflammatory cytokines, including interleukin-6 (IL-6) and tumor necrosis factor-alpha (TNF-α). The inflammatory responses contribute to the amplification of oxidative stress through the facilitation of the release of enzymes that produce reactive oxygen species (ROS) from immune cells, as well as the stimulation of NADPH oxidase expression [16,17]. The activation of the polyol pathway is initiated by hyperglycemia, which results in an increased flow of glucose through aldose reductase and subsequent accumulation of sorbitol. This particular process leads to the depletion of cellular NADPH and contributes to oxidative stress by compromising antioxidant defense mechanisms [16]. The impairment of nitric oxide (NO) bioavailability and the stimulation of adhesion molecule expression are promoted by oxidative stress, leading to endothelial dysfunction and atherosclerosis. This condition is known to contribute to the development of atherosclerosis, a significant cardiovascular complication commonly associated with diabetes [18]. Oxidative stress is known to play a significant role in the pathogenesis of diabetic neuropathy, leading to the impairment of peripheral nerves and subsequent neurodegeneration. It has the potential to interfere with nerve conduction, induce neuroinflammation, and contribute to neuronal apoptosis [19]. Oxidative stress plays a significant role in the development of diabetic nephropathy by facilitating damage to the glomerular and tubular structures within the kidneys. It facilitates the augmentation of extracellular matrix proteins and cytokines implicated in fibrosis and inflammation [20]. The complex relationship between elevated blood sugar levels (hyperglycemia) and the harmful effects of oxidative stress is a key factor in the development and advancement of different complications associated with diabetes. The strategic targeting of oxidative stress pathways has been identified as a promising therapeutic approach for mitigating the adverse effects of hyperglycemia. Strategies focused on restoring redox balance and augmenting antioxidant defense mechanisms show potential for mitigating the impact of complications associated with diabetes. In summary, it can be concluded that hyperglycemia-induced oxidative stress is an intricate and multifaceted phenomenon that plays a significant role in the development of diabetic complications. A thorough comprehension of these mechanisms offers valuable insights into potential therapeutic interventions aimed at mitigating the effects of diabetes on multiple organ systems.

Inflammation and Endothelial Dysfunction: Accelerated Atherosclerosis and Plaque Instability

Atherosclerosis is a multifaceted, persistent inflammatory condition distinguished by the buildup of lipids, immune cells, and extracellular matrix within the arterial wall, resulting in the formation of atherosclerotic plaques. The development of atherosclerosis entails a complex sequence of cellular and molecular processes. Recent research has shed light on the significant impact of inflammation and endothelial dysfunction in hastening atherosclerosis and fostering instability within plaques.

The relationship between inflammation and atherosclerosis: Inflammation plays a crucial role in the initiation and progression of atherosclerosis. The condition is initiated by various factors, including altered lipoproteins (such as oxidized LDL), cytokines, and infiltration of immune cells. Activated immune cells release inflammatory cytokines such as interleukin-1 (IL-1), IL-6, and tumor necrosis factor-alpha (TNF-α), which play a role in the recruitment of monocytes to the arterial wall [20,21]. The process of monocyte recruitment and foam cell formation involves the expression of adhesion molecules, such as vascular cell adhesion molecule-1 (VCAM-1) and intercellular adhesion molecule-1 (ICAM-1), by activated endothelial cells. These molecules play a crucial role in facilitating the adhesion and migration of monocytes into the subendothelial space. Upon entering the intima, monocytes undergo differentiation into macrophages and proceed to internalize modified lipoproteins, subsequently undergoing transformation into foam cells. The presence of foam cells is a characteristic feature observed in early atherosclerotic lesions [20]. The role of endothelial dysfunction is significant in the development of atherosclerosis, as it is characterized by a decrease in the availability of nitric oxide (NO) and an increase in the expression of endothelin-1. The production of nitric oxide (NO) by endothelial nitric oxide synthase (eNOS) is essential for the regulation of vascular tone and the prevention of platelet aggregation. The presence of dysfunctional endothelium contributes to the development of a pro-inflammatory environment and diminishes the anti-thrombotic characteristics of the vessel wall [22]. Oxidative stress and lipid peroxidation are significant factors in the development of endothelial dysfunction. This occurs as a result of an imbalance between the production of ROS and the body's antioxidant defense mechanisms. Oxidized LDL molecules elicit an inflammatory response in endothelial cells, thereby initiating the secretion of chemokines that recruit immune cells to the site of injury. In addition, the oxidation of lipids induces the production of ROS, thereby exacerbating inflammation and compromising endothelial function [20].

Plaque formation and instability: The atherosclerotic plaque undergoes a dynamic process of remodeling, resulting in a transition from stable to unstable plaques. Unstable plaques exhibit distinct features, including a thin fibrous cap, a substantial lipid-rich necrotic core, and an elevated presence of inflammatory cells. The process of plaque neovascularization and intraplaque hemorrhage, which are influenced by angiogenesis and inflammation, play a significant role in the destabilization of plaques. The rupture of the fibrous cap results in the exposure of thrombogenic material to the bloodstream. This exposure initiates platelet aggregation and the formation of a thrombus, which can ultimately lead to myocardial infarction or stroke [23].

Therapeutic implications: The comprehension of the complex relationship between inflammation, endothelial dysfunction, and atherosclerosis has resulted in the emergence of innovative therapeutic strategies. Statins, as such, have the ability to not only reduce cholesterol levels but also exhibit anti-inflammatory properties that contribute to the stabilization of plaques. Anti-inflammatory agents, such as canakinumab, that specifically target IL-1β have demonstrated potential for mitigating cardiovascular events. Furthermore, there is ongoing research on interventions aimed at enhancing endothelial function, including the investigation of angiotensin-converting enzyme inhibitors (ACEIs) and exercise [22]. In brief, there exists a close relationship between inflammation and endothelial dysfunction, which collectively contribute to the acceleration of atherosclerosis and the promotion of plaque instability. The interaction among immune cells, endothelial cells, and oxidized lipids establishes a microenvironment that promotes the development and rupture of plaques. The advancements in comprehending these mechanisms have paved the way for targeted therapeutic strategies that aim to effectively mitigate the impact of CVD.

Epidemiology of CVD in diabetes

Macrovascular Complications: Coronary Artery Disease (CAD) and Stroke

CVD is a significant contributor to both morbidity and mortality among individuals diagnosed with diabetes mellitus. The presence of diabetes greatly increases the likelihood of developing macrovascular complications, including CAD and stroke. The epidemiological data regarding these complications highlight the urgent requirement for efficient management and preventive strategies. CAD is a medical condition that affects the arteries supplying blood to the heart. Diabetes is widely recognized as a significant risk factor for CAD, a medical condition characterized by the constriction or obstruction of coronary arteries caused by the development of atherosclerotic plaques. Individuals with diabetes have a significantly higher risk of CAD compared to the general population. Based on findings from the Global Burden of Disease Study, it has been observed that CAD has significantly contributed to the number of deaths associated with diabetes on a global scale in recent times [24]. The coexistence of diabetes mellitus exacerbates CAD by hastening the progression of atherosclerosis, fostering the instability of plaque, and compromising endothelial function. These effects are attributed to various mechanisms, including inflammation, oxidative stress, and dyslipidemia. The presence of diabetes significantly increases the risk of both ischemic and hemorrhagic strokes. Ischemic strokes are a result of the occlusion of cerebral blood vessels, typically caused by atherosclerosis or emboli originating from the heart. Hemorrhagic strokes occur as a consequence of the rupture of blood vessels within the brain. According to the findings of the INTERSTROKE study, there is a significant correlation between diabetes and an elevated risk of stroke [22]. Inadequate management of diabetes can contribute to increased risk factors for stroke, such as hypertension and dyslipidemia.

Microvascular Complications: Diabetic Nephropathy and Retinopathy

Microvascular complications refer to a group of medical conditions that arise as a result of damage to the small blood vessels in the body. Diabetic nephropathy and retinopathy are two medical conditions commonly associated with diabetes.

Diabetic Nephropathy: Diabetic nephropathy is a microvascular complication that impacts renal function, resulting in gradual deterioration of kidney health and eventual renal failure. It is a frequently encountered etiology of end-stage renal disease (ESRD). The epidemiology of diabetic nephropathy underscores its significant implications for public health. The Diabetes Control and Complications Trial (DCCT) and the United Kingdom Prospective Diabetes Study (UKPDS) have provided substantial evidence regarding the significant correlation between glycemic control and the risk of diabetic nephropathy. Both type 1 and type 2 diabetes are associated with the development of nephropathy. Multiple factors, such as hyperglycemia, oxidative stress, and inflammation, contribute to its pathogenesis [24].

Diabetic retinopathy: It is a medical condition characterized by the involvement of the retina's microvasculature, potentially resulting in visual impairment or complete loss of vision. The incidence of diabetic retinopathy is strongly associated with the duration of diabetes and the degree of glycemic control. Based on findings from the Wisconsin Epidemiologic Study of Diabetic Retinopathy, it was observed that around 28.5% of individuals diagnosed with diabetes exhibited symptoms of diabetic retinopathy [25]. Diabetic retinopathy advances through distinct stages, namely non-proliferative and proliferative stages, characterized by varying levels of severity. The presence of chronic hyperglycemia and other metabolic abnormalities plays a significant role in the progression of retinal microvascular damage, ultimately leading to vision impairment. The epidemiology of cardiovascular complications in individuals with diabetes highlights the pressing necessity for comprehensive management strategies that effectively target both macrovascular and microvascular complications. The implementation of a comprehensive approach, which encompasses glycemic control, blood pressure management, lipid-lowering therapy, and lifestyle interventions, plays a pivotal role in mitigating the impact of CVD among individuals with diabetes. It is crucial to have a comprehensive understanding of the complex relationship between diabetes and its associated complications in order to develop successful strategies for prevention and treatment.

Preventive measures to reduce cardiovascular risk in diabetic patients

Glycemic Control and Its Impact on Cardiovascular Outcomes

The regulation of blood glucose levels, known as glycemic control, is a crucial component of diabetes management that has a profound impact on the overall well-being of individuals living with diabetes. The correlation between glycemic control and cardiovascular outcomes is a multifaceted interaction of various factors, encompassing both microvascular and macrovascular complications. Microvascular complications refer to a group of medical conditions that arise as a result of damage to the small blood vessels in the body. The implementation of intensive glycemic control has consistently shown positive outcomes in mitigating the risk of microvascular complications. The Diabetes Control and Complications Trial (DCCT) and the United Kingdom Prospective Diabetes Study (UKPDS) have yielded significant findings regarding the influence of glycemic control on diabetic retinopathy, nephropathy, and neuropathy [26,27]. The trials conducted have brought attention to the fact that there is a correlation between maintaining lower levels of HbA1c and a reduced risk of developing microvascular complications. The implementation of strict glycemic control has been shown to effectively decrease the activation of biochemical pathways associated with the development of advanced glycation end-products (AGEs). These AGEs are known to play a significant role in the progression of microvascular damage.

Macrovascular complications: The relationship between glycemic control and macrovascular outcomes, specifically CVD, is complex. Although hyperglycemia is widely acknowledged as a significant factor in the development of atherosclerosis and CVD, the connection between glycemic control and macrovascular outcomes is not as clear-cut as it is in the case of microvascular complications. The Action to Control Cardiovascular Risk in Diabetes (ACCORD) trial and the Veterans Affairs Diabetes Trial (VADT) have shed light on the intricate relationship between glycemic control and cardiovascular outcomes [28,29]. The purpose of these trials was to investigate the potential impact of intensive glycemic control, specifically targeting lower HbA1c levels, on the occurrence of cardiovascular events. Both trials yielded inconclusive results regarding the effectiveness of intensive glycemic control in reducing major cardiovascular events. Based on the findings, it can be inferred that the association between glycemic control and macrovascular outcomes may be impacted by various factors, including hypoglycemia, duration of diabetes, and pre-existing CVD. The notion of "glycemic memory" underscores the significance of initiating and maintaining glycemic control at an early stage. The Epidemiology of Diabetes Interventions and Complications (EDIC) trial, which is an extension of the DCCT, has shown that previous intensive glycemic control in the DCCT arm continues to provide protective effects against CVD, even after glycemic control has become equalized between the standard and intensive groups [27].

Individualized approach: The provision of personalized care is of utmost importance in achieving optimal glycemic control. The establishment of glycemic targets is recommended by the American Diabetes Association (ADA) and other guidelines, taking into consideration various factors such as age, comorbidities, life expectancy, and the risk of hypoglycemia [29]. For example, older adults who have multiple comorbidities may experience fewer complications related to hypoglycemia if less stringent glycemic targets are set. Maintaining glycemic control is a crucial component of managing diabetes, as it has significant implications for both microvascular and macrovascular complications. The relationship between intensive glycemic control and cardiovascular outcomes is multifaceted and influenced by numerous factors. However, it has been observed that initiating and maintaining glycemic control early on, along with personalized care, can substantially decrease the likelihood of long-term complications in individuals with diabetes.

Blood Pressure Management in Diabetic Patients

Hypertension is a prevalent and critical comorbidity in individuals with diabetes mellitus, significantly increasing their risk of cardiovascular events and complications. Effective blood pressure management is crucial in mitigating these risks and improving overall outcomes for diabetic patients.

Prevalence and impact: Hypertension is a common complication of diabetes, affecting a substantial proportion of individuals with both type 1 and type 2 diabetes. The National Health and Nutrition Examination Survey (NHANES) data indicated that approximately 71% of adults with diabetes had hypertension [28]. Hypertension amplifies the risk of CVD by promoting atherosclerosis, endothelial dysfunction, and left ventricular hypertrophy. Diabetic nephropathy and retinopathy are exacerbated by hypertension, making effective blood pressure management crucial.

Blood pressure targets: Blood pressure targets for diabetic patients have evolved over time. Historically, the Hypertension Optimal Treatment (HOT) trial and the UK Prospective Diabetes Study (UKPDS) recommended systolic blood pressure (SBP) targets of less than 150 mmHg. However, recent guidelines, including those from the American Diabetes Association (ADA) and the American College of Cardiology (ACC), advocate for more stringent blood pressure targets [29]. The Systolic Blood Pressure Intervention Trial (SPRINT) emphasized the benefits of intensive blood pressure control, targeting an SBP of less than 120 mmHg in high-risk individuals [30]. These findings prompted a shift in guidelines, recommending diabetic patients to aim for an SBP of less than 130 mmHg. The individualization of targets is essential, considering factors like age, comorbidities, and hypotension risk.

Management strategies: Lifestyle modifications are fundamental to blood pressure management. The Dietary Approaches to Stop Hypertension (DASH) diet, rich in fruits, vegetables, whole grains, lean proteins, and low-fat dairy, has been shown to effectively lower blood pressure. Sodium restriction, weight management, regular physical activity, and limited alcohol consumption complement lifestyle changes. Antihypertensive medications play a crucial role when lifestyle interventions are insufficient. Thiazide diuretics, ACEIs, angiotensin II receptor blockers (ARBs), calcium channel blockers, and beta-blockers are commonly used. In diabetic patients with albuminuria, ACEIs and ARBs are particularly beneficial in slowing the progression of diabetic nephropathy [31].

Combination therapy: The ACCORD trial highlighted the potential benefits of combination therapy. Diabetic patients often require multiple antihypertensive agents to achieve blood pressure targets. Combination therapy offers synergistic effects, reduced pill burden, and improved adherence. However, the choice of medications should consider potential side effects, such as hypotension and renal function decline. Blood pressure management is a cornerstone of diabetes care, significantly impacting cardiovascular outcomes and complications. The evolving landscape of blood pressure targets, informed by landmark trials like SPRINT, underscores the importance of individualized care. Lifestyle modifications and judicious use of antihypertensive medications, in alignment with guidelines, are vital components of comprehensive blood pressure management for diabetic patients.

Lipid-Lowering Therapy and Its Role in Cardiovascular Risk Reduction

Lipid-lowering therapy is of paramount importance in mitigating cardiovascular risk, particularly in individuals diagnosed with diabetes mellitus, who exhibit a higher susceptibility to dyslipidemia and atherosclerosis. Increased levels of LDL-C and triglycerides, combined with decreased HDL-C, contribute to the development of atherosclerosis. Statins are a class of medications that effectively inhibit the synthesis of cholesterol, making them a fundamental component of lipid-lowering therapy. Extensive evidence has demonstrated the significant benefits of statins in reducing cardiovascular risk. According to the meta-analysis conducted by the Cholesterol Treatment Trialists' (CTT), there is a significant correlation between the reduction of LDL-C levels and a proportional decrease of approximately 21% in major vascular events (Cholesterol Treatment Trialists' Collaboration et al., 2010). It is worth noting that individuals with diabetes can potentially experience similar, if not more significant, benefits from statin therapy. The findings of the Collaborative Atorvastatin Diabetes Study (CARDS) indicate that the use of atorvastatin resulted in a significant reduction in the occurrence of major cardiovascular events among individuals with diabetes [32]. When statins alone fail to achieve optimal lipid levels, additional agents such as ezetimibe and proprotein convertase subtilisin/kexin type 9 (PCSK9) inhibitors may be taken into consideration. Ezetimibe effectively inhibits the absorption of cholesterol in the intestines, whereas PCSK9 inhibitors have the ability to enhance the recycling of LDL receptors. This mechanism ultimately results in an increased clearance of LDL-C from the bloodstream. These agents have demonstrated additional advantages in effectively lowering LDL-C levels, particularly in patients diagnosed with familial hypercholesterolemia or those who experience intolerance to statins.

Lifestyle Interventions: Recommendations for Diet and Exercise

Lifestyle interventions play a fundamental role in the management of cardiovascular risk factors in individuals diagnosed with diabetes. Dietary modifications and regular exercise play a crucial role in achieving optimal glycemic control, effectively managing blood pressure, and facilitating weight reduction. The DASH diet places emphasis on the consumption of nutrient-rich foods, including whole grains, fruits, vegetables, lean proteins, and low-fat dairy products [33]. This dietary regimen has been found to be highly effective in lowering blood pressure levels and enhancing lipid profiles. Furthermore, scientific studies have demonstrated that the Mediterranean diet, which is abundant in beneficial fats such as olive oil and omega-3 fatty acids, exhibits cardioprotective properties. Both dietary patterns are effective in promoting weight loss and improving metabolic health. Regular physical activity is equally essential. Aerobic exercises such as brisk walking, jogging, or swimming have been shown to improve cardiovascular fitness and increase insulin sensitivity. Resistance training has been shown to enhance muscle mass and play a significant role in promoting overall metabolic health [33,34]. The Look AHEAD (Action for Health in Diabetes) trial findings indicated that lifestyle interventions incorporating dietary modifications and physical activity resulted in significant weight reduction and enhancements in cardiovascular risk factors for individuals diagnosed with type 2 diabetes [35].

Smoking Cessation and Its Impact on Cardiovascular Risk

The act of smoking is a significant modifiable risk factor that greatly increases the likelihood of developing CVD. This risk is further amplified for individuals who have diabetes. The act of smoking has been found to expedite the progression of atherosclerosis, contribute to the impairment of endothelial function, and elevate the likelihood of thrombosis. It worsens the pro-inflammatory and pro-thrombotic condition linked to diabetes. The cessation of smoking is of utmost importance in the effort to decrease cardiovascular risk. The UK Prospective Diabetes Study (UKPDS) demonstrated that cessation of smoking was associated with a significant reduction in the risk of myocardial infarction among individuals diagnosed with diabetes (UK Prospective Diabetes Study Group, 1998). Behavioral counseling, pharmacotherapy, and support groups are all essential components in supporting individuals with diabetes in their endeavors to cease smoking [33]. Lipid-lowering therapy, lifestyle interventions, and smoking cessation are essential elements of comprehensive cardiovascular risk reduction strategies for individuals diagnosed with diabetes. The integration of medical therapy with healthy lifestyle choices presents a synergistic approach that effectively tackles the multifaceted cardiovascular risk factors prevalent in this high-risk population. By implementing these strategies, healthcare providers have the potential to greatly improve the overall health and well-being of individuals who are living with diabetes.

Treatment options for diabetic patients with CVD

Antiplatelet Therapy: Aspirin and Beyond

Antiplatelet therapy is a cornerstone in managing CVD in diabetic patients. Aspirin, a widely used antiplatelet agent, reduces the risk of cardiovascular events by inhibiting platelet aggregation. The Antiplatelet Trialists' Collaboration meta-analysis showed that aspirin reduced the risk of major vascular events by about 25% in patients with a history of vascular disease [36]. However, recent guidelines, including those from the American Diabetes Association (ADA) and the American College of Cardiology (ACC), have nuanced recommendations on aspirin use. The benefit-risk balance of aspirin varies depending on the individual's age, bleeding risk, and history of CVD. Emerging evidence suggests that the net benefit of aspirin for primary prevention in diabetic patients without established CVD may be limited.

ACEIs and ARBs

ACEIs and ARBs are integral in the management of diabetic patients with CVD. These agents have shown substantial benefits in reducing morbidity and mortality. They exert cardioprotective effects by inhibiting the renin-angiotensin-aldosterone system, reducing blood pressure, and attenuating adverse cardiac remodeling. The Heart Outcomes Prevention Evaluation (HOPE) trial demonstrated that ramipril, an ACEI, significantly reduced cardiovascular events in high-risk patients, including those with diabetes [37]. ARBs like losartan have shown similar benefits in diabetic patients, as seen in the Irbesartan in Patients with Type 2 Diabetes and Microalbuminuria (IRMA-2) trial [38].

Sodium-Glucose Cotransporter-2 (SGLT2) Inhibitors

SGLT2 inhibitors are a breakthrough class of medications that have revolutionized the treatment landscape for diabetic patients with CVD. These agents not only lower blood glucose levels by promoting urinary glucose excretion but also offer remarkable cardiovascular benefits. The Empagliflozin Cardiovascular Outcome Event Trial in Type 2 Diabetes Mellitus Patients (EMPA-REG OUTCOME) trial demonstrated that empagliflozin significantly reduced the risk of major adverse cardiovascular events and cardiovascular death in diabetic patients with established CVD [34]. Similarly, the Canagliflozin Cardiovascular Assessment Study (CANVAS) showed cardiovascular risk reduction with canagliflozin [35]. The mechanisms underlying these benefits include improvements in heart failure outcomes, blood pressure reduction, and potential metabolic effects.

Glucagon-Like Peptide-1 (GLP-1) Receptor Agonists

GLP-1 receptor agonists are another class of medications offering cardiovascular benefits in diabetic patients. These agents enhance glucose-dependent insulin secretion, suppress glucagon secretion, delay gastric emptying, and promote satiety. The Liraglutide Effect and Action in Diabetes: Evaluation of Cardiovascular Outcome Results (LEADER) trial demonstrated that liraglutide reduced the risk of major cardiovascular events and cardiovascular death in patients with type 2 diabetes and high cardiovascular risk [38]. Similarly, the Semaglutide Cardiovascular Outcomes Trial (SUSTAIN-6) showed cardiovascular benefits with semaglutide [37].

Novel Therapies and Future Directions

Research into novel therapies for diabetic patients with CVD is ongoing. Therapies targeting inflammation, lipid metabolism, and other pathways are being investigated. The Canakinumab Anti-inflammatory Thrombosis Outcome Study (CANTOS) trial explored the use of canakinumab, an anti-inflammatory agent, in reducing cardiovascular events [39]. Personalized medicine, guided by genetic and molecular insights, holds promise in tailoring treatment strategies. Precision medicine could identify subgroups of patients who would benefit most from specific interventions, optimizing outcomes. The landscape of treatment options for diabetic patients with CVD is rapidly evolving. Antiplatelet therapy, ACEIs, ARBs, SGLT2 inhibitors, and GLP-1 receptor agonists have demonstrated substantial cardiovascular benefits beyond glycemic control. These agents offer multifaceted mechanisms of action, including blood pressure reduction, anti-inflammatory effects, and metabolic improvements. Novel therapies and personalized medicine approaches further expand the horizon for improving cardiovascular outcomes in this high-risk population.

Multidisciplinary approach to cardiovascular risk reduction

Importance of Team-Based Care

CVD continues to be a prominent cause of illness and death among individuals diagnosed with diabetes. In light of the intricate interplay of various risk factors and the complex nature of cardiovascular complications associated with diabetes, it is crucial to adopt a comprehensive and integrated approach. The implementation of a multidisciplinary team-based care model is recognized as a crucial strategy for effectively addressing cardiovascular risk in this population at high risk. The multifaceted nature of cardiovascular complications. Diabetes is a chronic condition that impacts multiple organ systems, and it is associated with notable cardiovascular complications. These complications involve a range of issues, such as atherosclerosis, endothelial dysfunction, hypertension, dyslipidemia, and heart failure. Each of these factors contributes to the increased cardiovascular risk that individuals with diabetes encounter. A multidisciplinary team-based care model recognizes that no single healthcare professional possesses the expertise to comprehensively manage the myriad facets of cardiovascular risk in diabetes. The process entails the collaboration of various specialists, each leveraging their distinct expertise and abilities to address specific facets of the patient's well-being.

Efforts coordination: The multidisciplinary team consists of endocrinologists, cardiologists, primary care providers, nurses, dietitians, exercise physiologists, and behavioral psychologists. These experts work together to develop a personalized treatment plan that takes into account the patient's medical history, current health condition, as well as their lifestyle, preferences, and objectives. In order to comprehensively address all potential risk factors, the multidisciplinary team conducts a methodical evaluation of the patient's individual risk factors. Endocrinologists are responsible for the management of glycemic control, while cardiologists focus on addressing pre-existing cardiovascular conditions. Primary care providers play a crucial role in monitoring overall health, while nurses provide ongoing support and monitoring. Dietitians offer valuable nutritional guidance, and exercise physiologists are involved in designing fitness plans. Lastly, behavioral psychologists assist individuals in making lifestyle changes and ensuring adherence to recommended treatments.

Optimization of treatment strategies: The collaboration among healthcare professionals cultivates an atmosphere in which treatment strategies are maximized. For example, the medication regimen of a patient is carefully coordinated to prevent any potential conflicts or duplications. The input and recommendations provided by a specialist are taken into consideration by other professionals in order to develop a comprehensive and integrated care plan. Holistic patient-centered care is an approach that recognizes the individuality of individuals with diabetes, taking into account their unique needs and challenges. This approach involves a multidisciplinary team that collaborates to provide comprehensive care. By adopting a patient-centered approach, our team takes into account not only the medical aspects but also factors such as cultural background, socioeconomic status, and psychological well-being. This promotes increased patient engagement, satisfaction, and adherence to treatment plans. Improved patient outcomes have been demonstrated through the implementation of a multidisciplinary team-based care approach, as indicated by various studies. The Look AHEAD trial provided evidence that intensive lifestyle interventions, under the guidance of a multidisciplinary team, resulted in significant improvements in cardiovascular risk factors among individuals with diabetes [38]. The team's collaborative efforts are expected to yield improvements in glycemic control, blood pressure management, lipid profiles, and overall cardiovascular health. The reduction of cardiovascular risk in individuals with diabetes requires a collaborative and coordinated approach from healthcare professionals in various disciplines. The multidisciplinary team-based care model acknowledges the complex nature of cardiovascular complications associated with diabetes and ensures that patients receive comprehensive care that addresses multiple risk factors. By engaging in collaborative efforts and prioritizing the unique requirements of every patient, healthcare teams have the potential to greatly enhance cardiovascular outcomes and enhance the overall quality of life for individuals who are living with diabetes.

Role of Cardiologists, Endocrinologists, and Primary Care Providers

Each healthcare professional within the multidisciplinary team brings a unique skill set to the table, contributing to the holistic management of cardiovascular risk.

Cardiologists: Cardiologists are pivotal in evaluating and managing CVD in diabetic patients. They perform risk assessments, diagnostic tests, and interventions such as coronary angiography and percutaneous coronary intervention. Cardiologists guide the use of medications like statins, antiplatelet agents, and ACEIs to address cardiovascular risk factors.

Endocrinologists: Endocrinologists are central to glycemic control, a key aspect of cardiovascular risk reduction. They manage diabetes medications, including insulin, and tailor treatment plans based on blood glucose levels, HbA1c targets, and individual patient characteristics. Endocrinologists also coordinate with other specialists to ensure comprehensive care.

Primary Care Providers (PCPs): PCPs play a crucial role in coordinating overall care. They monitor patients' general health, provide routine check-ups, and identify potential risk factors. PCPs often initiate lifestyle interventions, prescribe medications, and refer patients to specialists when needed. They serve as a bridge between different specialists, ensuring seamless communication and care coordination.

Patient Education and Empowerment

In the multidisciplinary approach to cardiovascular risk reduction for individuals with diabetes, patient education and empowerment emerge as crucial components that drive informed decision-making, active engagement, and ultimately, improved cardiovascular outcomes. Patient education is the foundation upon which individuals can understand the complexities of diabetes and its implications for cardiovascular health. Healthcare professionals play a pivotal role in imparting accurate and comprehensive information to patients.

Diabetes and cardiovascular implications: Patients need to comprehend the strong link between diabetes and CVD. They should be educated about how elevated blood sugar levels contribute to atherosclerosis, endothelial dysfunction, and other cardiovascular complications.

Risk reduction strategies: Healthcare providers explain strategies for reducing cardiovascular risk, which encompass not only glycemic control but also blood pressure management, cholesterol levels, and lifestyle modifications. Patients learn how these risk factors interplay and impact their overall health.

Empowerment: Empowering patients involves equipping them with the knowledge and skills necessary to actively participate in their own care and decision-making process. This empowerment fosters a sense of ownership and responsibility for their health.

Informed decision-making: Patients are educated about various treatment options, medications, and interventions available to manage diabetes and cardiovascular risk. This empowers them to make informed choices based on their preferences and values.

Self-management skills: Patients are taught practical skills, such as monitoring blood glucose levels, adhering to medications, and recognizing symptoms of hypo- or hyperglycemia. These skills enable patients to take charge of their day-to-day management.

Lifestyle modifications: Patients receive guidance on adopting a heart-healthy lifestyle, including dietary changes, regular physical activity, smoking cessation, and stress management. By understanding the significance of these changes, patients are more likely to implement and sustain them.

Shared decision-making: Shared decision-making emphasizes collaboration between patients and healthcare professionals, recognizing that patients' values, preferences, and goals are central to their care.

Patient preferences: Patients are encouraged to express their preferences, concerns, and aspirations regarding their health. This active involvement fosters a sense of partnership and mutual respect.

Expert guidance: Healthcare professionals offer their expertise and evidence-based guidance, presenting patients with the pros and cons of different interventions. This collaborative approach ensures that treatment plans align with the patient's individual context.

Enhanced adherence: By actively participating in decisions about their care, patients are more likely to adhere to treatment plans. This contributes to improved long-term outcomes and better disease management.

Evidence of effectiveness: The efficacy of patient education and empowerment within a multidisciplinary approach is underscored by studies like the Steno-2 study. This study demonstrated that addressing multiple risk factors through comprehensive care led to a significant reduction in cardiovascular morbidity and mortality in individuals with diabetes [38].

Patient education and empowerment lie at the heart of the multidisciplinary approach to cardiovascular risk reduction in individuals with diabetes. By providing patients with the knowledge, skills, and tools to actively engage in their care, healthcare professionals foster a collaborative environment that optimizes cardiovascular outcomes. Through shared decision-making, patients become partners in their journey toward better health, ensuring that interventions are aligned with their needs and preferences. By embracing this model, healthcare systems can create a profound positive impact on the long-term cardiovascular health and overall well-being of individuals living with diabetes.

Challenges and future perspectives

Addressing Health Disparities in Diabetes and CVD

The presence of health disparities in diabetes and CVD highlights the significance of implementing focused interventions to guarantee fair and unbiased healthcare for individuals, irrespective of socioeconomic, ethnic, or geographic variables.

Overview of health disparities: Certain demographic groups, such as racial and ethnic minorities and individuals with lower socioeconomic status, exhibit elevated prevalence rates of diabetes and its related cardiovascular complications. Disparities in access to healthcare, economic resources, and education are significant contributing factors to these inequities.

Challenges: Insufficient availability of healthcare services, such as preventive screenings and specialized consultations, can result in delayed diagnosis and suboptimal management of diabetes and CVD. Cultural and linguistic barriers may present obstacles to effective communication and comprehension between healthcare providers and patients, potentially impacting treatment adherence and overall health outcomes. Various socioeconomic factors, including poverty, food insecurity, and inadequate neighborhood safety, have the potential to significantly impact health behaviors and outcomes. It is imperative to address these determinants in order to achieve optimal reduction of cardiovascular risk.

Future perspectives: It is imperative to prioritize tailored interventions that take into account cultural, linguistic, and socioeconomic factors. Community health workers, possessing a comprehensive understanding of the distinct requirements of marginalized populations, are instrumental in providing culturally sensitive education and assistance. The utilization of telehealth and mobile clinics has the potential to enhance healthcare accessibility in remote or underserved regions. Telemedicine offers a robust platform for conducting virtual consultations and monitoring, effectively mitigating the impact of geographical barriers. Educational endeavors that prioritize health literacy have the potential to empower individuals by equipping them with the knowledge and understanding necessary to comprehend their medical conditions, evaluate treatment options, and implement effective self-care strategies.

Adherence to Medications and Lifestyle Interventions

The consistent adherence to medications and lifestyle interventions continues to present an ongoing challenge in effectively managing diabetes and cardiovascular risk. The management of diabetes frequently necessitates the use of multiple medications, each with its own unique dosing schedules and instructions. In conjunction with modifications to one's lifestyle, the intricacy of this situation can potentially overwhelm patients and result in a lack of adherence. The adoption and maintenance of healthy behaviors, such as engaging in regular exercise and making dietary modifications, necessitate a consistent level of motivation and a commitment to behavioral change. Numerous individuals encounter challenges in maintaining consistency. Customizing treatment plans based on individual preferences and capabilities has the potential to improve adherence. Healthcare professionals have the opportunity to engage in collaborative efforts with patients to establish attainable objectives and develop actionable plans. Mobile applications, wearable devices, and remote monitoring tools have the capability to deliver timely reminders, monitor progress, and provide immediate support. These technologies have the potential to promote accountability and engagement. By integrating behavioral psychology techniques, such as cognitive-behavioral therapy, it is possible to effectively address the psychological factors associated with behavior change and enhance adherence.

Integrating Digital Health Solutions in Diabetes Management

The incorporation of digital health solutions presents a significant potential for transforming the management of diabetes and cardiovascular risk. Applications provide a range of functionalities including glucose monitoring, medication notifications, meal preparation assistance, and exercise recommendations. Certain applications also enable remote communication with healthcare providers. Wearable devices such as continuous glucose monitors and fitness trackers offer real-time data, empowering users to actively monitor and effectively manage their health. Virtual consultations facilitate the connection between patients and healthcare professionals, enabling them to conduct check-ups, make necessary medication adjustments, and provide educational support, all without the requirement of in-person visits. The acquisition and dissemination of health data give rise to concerns regarding patient confidentiality and the safeguarding of data integrity. There exists a segment of the population that lacks access to smartphones, computers, or reliable internet connections, thereby constraining the potential reach of digital health solutions [39]. The utilization of advanced analytics has the potential to convert unprocessed data into practical insights, enabling users to receive tailored recommendations for effectively managing their conditions. Digital solutions facilitate the ongoing monitoring of essential parameters, enabling healthcare providers to promptly intervene in the event of abnormal readings. Interactive platforms have the capability to provide behavioral interventions, thereby facilitating the development of healthy habits and encouraging individuals to adhere to their treatment plans. In order to ensure the future success of cardiovascular risk reduction in diabetes, it is crucial to address health disparities, enhance adherence, and embrace digital health solutions [40]. By customizing interventions to cater to diverse populations, utilizing technology to provide personalized care, and encouraging active patient engagement, healthcare systems can address current obstacles and establish a more fair, efficient, and patient-centric approach to managing cardiovascular risks associated with diabetes.

Summary of the diabetes-CVD relationship

This narrative review aims to explore the complex relationship between diabetes and CVD, highlighting the significant interplay between these two conditions. The complex nature of cardiovascular complications associated with diabetes, which include issues such as endothelial dysfunction and accelerated atherosclerosis, highlights the pressing need for comprehensive strategies to reduce cardiovascular risk in individuals with diabetes. By conducting a comprehensive examination of inflammation, endothelial dysfunction, and accelerated atherosclerosis, we have successfully elucidated the complex mechanisms that contribute to the increased risk of CVD in individuals with diabetes. The findings obtained from this review have substantial implications for both clinical practice and public health initiatives. It is clear that a change in perspective is necessary when addressing cardiovascular risk reduction in individuals with diabetes. The implementation of the multidisciplinary model has proven to be an effective strategy in healthcare. This model involves the collaboration of various medical professionals, such as cardiologists, endocrinologists, primary care providers, and others, to develop a comprehensive care plan that is specifically tailored to meet the individual needs of each patient. The prioritization of patient education and empowerment is paramount, as it facilitates collaborative decision-making and cultivates a sense of personal accountability for one's well-being. In addition, it is crucial to prioritize the reduction of health disparities, improve medication adherence, and incorporate digital health solutions as essential approaches to achieve equitable healthcare and foster active patient participation. As we explore the future landscape of diabetes and cardiovascular risk reduction, there are several notable avenues for research and intervention that warrant attention. The investigation of personalized medicine approaches, utilizing genetics and biomarkers, shows potential for customizing interventions based on individual risk profiles. The incorporation of artificial intelligence and machine learning has the potential to enhance risk prediction models and treatment recommendations. The challenge of bridging the gap between clinical research and real-world implementation persists, necessitating the development of innovative strategies to effectively translate evidence into practice. The collaboration of researchers, healthcare providers, policymakers, and patients plays a crucial role in influencing the direction of diabetes and CVD management.

Limitations

The current narrative review, titled "Examining the Complex Relationship Between Diabetes and Cardiovascular Disease: Analyzing Approaches to Minimize Cardiovascular Risk in Patients With Diabetes," thoroughly summarizes the intricate connection between diabetes and CVD and explores methods aimed at mitigating cardiovascular risk in individuals with diabetes. Nevertheless, it is essential to acknowledge that this review has certain limitations that should be considered. To begin with, it is essential to acknowledge that selecting articles for inclusion in this narrative review may introduce a certain degree of selection bias. Although we employed a thorough search methodology by utilizing esteemed academic databases and search engines such as PubMed, MEDLINE, Embase, and Google Scholar, it is essential to acknowledge the possibility that some pertinent studies may have been unintentionally excluded. This could affect the overall comprehensiveness of the findings.

Furthermore, the diverse nature of the studies included in this review poses a significant challenge. The presence of various study methodologies, participant demographics, intervention strategies, and outcome measures may need to be addressed in reaching consistent conclusions across the existing body of literature. The presence of diverse elements necessitates caution when consolidating findings into comprehensive recommendations. In addition, it is essential to consider the issue of publication bias, a prevalent concern in reviews of this kind. It could distort the portrayal of study results. Research studies that demonstrate statistically significant effects or support specific interventions are more likely to be published. This can lead to an overrepresentation of favorable outcomes and a potential underrepresentation of studies with neutral or unfavorable findings.

Furthermore, the review's scope is constrained by its dependence on pre-existing studies, and the lack of original research could potentially limit the thoroughness of the analysis and the capacity to delve into intricacies within the data. The interpretation of study findings in the review is influenced by the perspectives of the reviewers and the potential for bias, which can impact the conclusions reached. Finally, it is essential to note that this study, being a narrative review, may need help conclusively establishing a causal relationship between specific interventions and outcomes. This is primarily due to the predominantly observational nature of the studies included in the analysis. Although attempts have been made to extract pertinent insights, the issue of causality remains intricate and necessitates additional investigation through experimental designs.

The narrative review "Exploring the Complex Connection Between Diabetes and Cardiovascular Disease: Analyzing Approaches to Mitigate Cardiovascular Risk in Patients With Diabetes" provides valuable insights. However, it is essential to exercise caution when interpreting the findings due to the limitations identified. Additional research, such as systematic reviews and meta-analyses, can enhance our comprehension of the topic, addressing certain limitations in this narrative review.

## Conclusions

This narrative review provides a comprehensive examination of the complex connection between diabetes and CVD. It delves into the impact of inflammation, endothelial dysfunction, and accelerated atherosclerosis on the increased risk of CVD. Highlighting the importance of adopting a multidisciplinary approach, the collaborative endeavors of healthcare professionals from various disciplines aim to customize care plans based on individual characteristics, thereby maximizing the effectiveness of patient-centered management. Patient education and empowerment are fundamental elements in achieving effective cardiovascular risk reduction. These elements facilitate informed participation in health management by promoting shared decision-making and enhancing adherence to treatment plans. The integration of scientific knowledge and patient-centered care presents an exciting opportunity for innovative approaches, including enhanced risk prediction models, tailored interventions, and effective research implementation. By utilizing this combination, healthcare providers have the opportunity to transform cardiovascular risk reduction, thereby promoting a more promising future for individuals with diabetes. This can be achieved through collaborative efforts encompassing research, clinical practice, policy, and patient engagement. This review highlights the potential of employing a multidisciplinary approach that combines scientific advancements with compassionate care. Such an approach has the capacity to improve outcomes and overall well-being for individuals living with diabetes.
